# Diabetic cardiomyopathy: Clinical phenotype and practice

**DOI:** 10.3389/fendo.2022.1032268

**Published:** 2022-12-07

**Authors:** Xudong Zhao, Shengwang Liu, Xiao Wang, Yibing Chen, Pai Pang, Qianjing Yang, Jingyi Lin, Shuaishuai Deng, Shentao Wu, Guanwei Fan, Bin Wang

**Affiliations:** ^1^ Department of Endocrine and Metabolic Diseases, First Teaching Hospital of Tianjin University of Traditional Chinese Medicine, Xiqing, Tianjin, China; ^2^ Tianjin Key Laboratory of Translational Research of TCM Prescription and Syndrome, First Teaching Hospital of Tianjin University of Traditional Chinese Medicine, Xiqing, Tianjin, China; ^3^ National Clinical Research Center for Chinese Medicine Acupuncture and Moxibustion, Xiqing, Tianjin, China

**Keywords:** diabetic cardiomyopathy, phase of progression, screening, diagnosis, treatment, review

## Abstract

Diabetic cardiomyopathy (DCM) is a pathophysiological condition of cardiac structure and function changes in diabetic patients without coronary artery disease, hypertension, and other types of heart diseases. DCM is not uncommon in people with diabetes, which increases the risk of heart failure. However, the treatment is scarce, and the prognosis is poor. Since 1972, one clinical study after another on DCM has been conducted. However, the complex phenotype of DCM still has not been fully revealed. This dilemma hinders the pace of understanding the essence of DCM and makes it difficult to carry out penetrating clinical or basic research. This review summarizes the literature on DCM over the last 40 years and discusses the overall perspective of DCM, phase of progression, potential clinical indicators, diagnostic and screening criteria, and related randomized controlled trials to understand DCM better.

## Introduction

The current diabetes mellitus (DM) prevalence is 463 million, 9.3% of the world population (1). In this vast group, heart failure (HF) has emerged as the most common cardiovascular complication of diabetes (2). Meanwhile, patients with type 2 diabetes mellitus (T2D) are more likely to be hospitalized and re-admitted for HF and have a higher risk of cardiovascular and all-cause mortality than those without diabetes and HF (3, 4); this may be due to long-term DM leading to pathological changes that contribute to the development and progression of HF, including myocardial structural, functional, and metabolic changes (5), independent of myocardial ischemia or atherosclerotic disease processes.

This distinct clinical entity was first proposed by Lundbaek (6) in 1954 as diabetic heart disease independent of hypertension and coronary artery disease (CAD) that commonly coexist with T2D. In 1972, the existence of DCM had been confirmed through postmortem pathological findings in four patients with diabetes who manifested HF symptoms, and DCM became validated as a distinct entity (2). Bertoni et al. (7) conducted a large nationwide case-control study in the United States in 1995, which confirmed an association between nonischemic idiopathic cardiomyopathy and diabetes. After these initial studies, DCM gained increased attention from epidemiologists and clinicians.

Despite the rapid increase in the number of preclinical and clinical studies on diabetic cardiomyopathy in the past decades, the process of DCM remains unclear. As a result, no consensus has been reached regarding the most effective preventive or therapeutic approaches to diabetic cardiomyopathy. For this reason, this review summarizes the recent theory and clinical finding achievements to understand this controversial disease better.

## Phases of progression

Diabetic cardiomyopathy is organic heart disease. The exact evolution of DCM pathological changes has not been fully studied. The existing Spatio-temporal evolution models of DCM are generally divided into two categories. One, which is the traditional understanding, is that DCM has only one phenotype, from diastolic to systolic dysfunction, and is accompanied by structural remodeling such as left ventricular hypertrophy (LVH), which is a gradual development process. The other believes that DCM is a disease with two independent phenotypes. The diastolic dysfunction phenotype eventually develops into HFpEF, and the systolic dysfunction phenotype finally develops into HFrEF, which is a new understanding.

**Figure 1:**
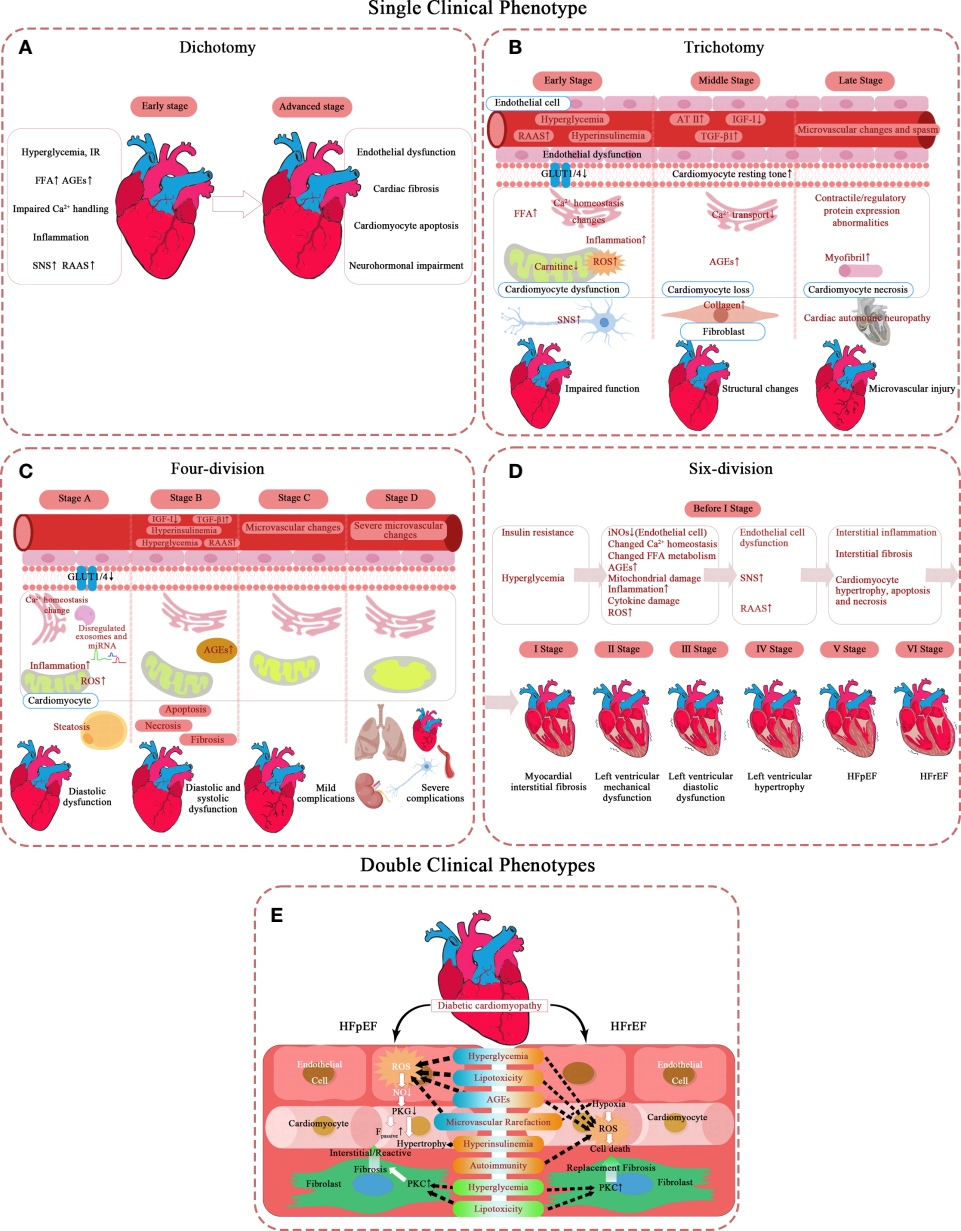
Pathological mechanism of DCM in different types of phases of progression. **(A–D)** Single clinical phenotype. **(E)** Double clinical phenotypes. AT-II, angiotensin II; AGEs, advanced glycation end-products; F_passive_, cardiomyocyte resting tension; FFA, free fatty acid; GLUT1, glucose transporter 1; GLUT4, glucose transporter 4; HFrEF, heart failure with reduced ejection fraction; HFpEF, heart failure with preserved ejection fraction; IR, insulin resistance; IGF-I, insulin-like growth factor 1; NO, nitric oxide; PKC, protein kinase C; PKG, protein kinase G; RAAS, renin-angiotensin-aldosterone system; ROS, reactive oxygen species; SNS, sympathetic nervous system; TGF-β1, transforming growth factor β1.

Specifically, five specific Spatio-temporal models have been proposed, and their pathological mechanisms have been briefly summarized (**Figure 1**). Moreover, a hypothesis is argued: there may be subclinical hyperfunction in the ultra-early stage of DCM.

### Single clinical phenotype

#### Dichotomy

The simplest model describes DCM as two stages, mainly in the left ventricle (8). The first is the stage of diastolic dysfunction, which is asymptomatic, but has a series of characteristics of ultrasonic cardiogram (9): elevated LV end-diastolic pressure, increased ventricular stiffness, and possibly accompanied by left ventricular concentric hypertrophy and left atrial enlargement.

The second stage of HF displays clinical symptoms and signs based on systolic dysfunction and severe cardiac remodeling, such as LVH and terminal LV dilatation.

#### Trichotomy

Scholars who hold the trisection view believe diabetic cardiomyopathy successively involves cardiac function – cardiac structure – microvascular (10–20). It takes years to induce significant LV dysfunction by gradually accumulating subcellular structural damage at the beginning (10). Diastolic dysfunction is the earliest clinical abnormality, with myocardial fibrosis and hypertrophy as the main reason. Systolic dysfunction only develops in the late stage of the disease (10) and often coexists with severe CAD and cardiac autonomic neuropathy (CAN) (11).

The early stage lasts only a short time. The entire initial stage is completely asymptomatic (12). At the very beginning, hyperglycemia and insulin resistance (IR) of DM patients have led the metabolic disorders in their hearts (21). At this stage, the heart structure is close to normal as a result of the compensatory adaptation of the heart to metabolic disorders, and only changes in myocardial cell substructure and endothelial dysfunction are observed (22–24). First, the level of GLUT4 on myocardial cell membrane decreases (25), and the activity of PPARα increases (26), which decreases the level of intracellular glucose oxidation (GLOX) (27). On the other hand, the signal transduction mediated by insulin receptor increases (28), which promotes the transport of fatty acid transferase (FAT/CD36) to the plasma membrane (29), and then increases the uptake of FA and the level of fatty acid oxidation (FAO). These unbalanced substrate metabolisms reduce the efficiency of myocardial ATP production, so that work efficiency of cardiomyocytes deteriorates. Discrete subclinical diastolic dysfunction can be detected clinically. The initial characteristics of DCM are increased atrial filling, decreased ventricular early diastolic filling, and increased myocardial relaxation damage and stiffness (10). The ventricular filling reduction is characterized by a slow E acceleration peak, deceleration peak, and peak filling rate, which can be captured by magnetic resonance imaging (30). Echocardiographic findings of ventricular septal annual wall motion damage also confirmed this (13). It is worth mentioning that the decrease of myocardial blood flow reserve can be identified by load imaging technology (31), which may be related to impaired insulin signal transduction (14).

In the middle stage, the aggravation of myocardial cell injury makes diastolic cardiac function significantly abnormal, but the ejection function is only slightly affected (EF 40%-50%) (15). With the progress of the disease, the effects of metabolic disorders gradually expand, in which dysfunctional mitochondria is a key role (32). The increase of FAO and the decrease of GLOX lead to the accumulation of toxic lipid metabolites, like DAG and ceramide, and the increase of oxidative stress, especially the release of ROS and NOS in cardiomyocytes (33, 34). Lipotoxicity caused by toxic lipid metabolites can lead to the remodeling of mitochondrial membrane, and oxidative stress can damage the proteins involved in oxidative phosphorylation (35) and activate uncoupling protein (UCP) (36), further destroying the function of mitochondria. In addition, under the influence of hyperglycemia, the levels of advanced glycation end products (AGE) increases both inside and outside the cells, and their formation on the SERCA2a and Ryr of the sarcoplasmic reticulum interfered with the dynamics of Ca2+ (37, 38), thus affecting the function of mitochondria (39). Mitochondria plays as a mediator in this process, which activates the apoptotic cascades under the influence of abovementioned factors (40). In short, the subcellular mechanisms, such as impaired insulin signal transduction, mitochondria metabolic disorders and impaired calcium dynamics, lead to cardiomyocyte loss and fibrosis, and may lead to mild CAN (15). This stage begins to show clinical symptoms. Although the change in cardiac structure is still slight, it can be detected by conventional echocardiography: LV diameter, wall thickness, or mass increases, and compliance decreases (14). Myocardial microvascular structural damage is not obvious at this stage.

This stage is a critical period of fibrosis progression because cardiac magnetic resonance (CMR) can detect diffuse fibrosis (16). The increase of perivascular and myofibrillar interstitial fibrosis was observed in the myocardial samples without coronary heart disease and hypertension (41). Stiff collagen and its crosslinks accumulate in the heart interstitial, causing the gradual loss of muscle cells. Notably, this collagen deposition and accumulation of advanced glycation end products (AGEs) are important features of increased LV stiffness in heart failure patients with reduced EF, which may be related to the impairment of ejection function (41).

In the last stage, cardiac interstitial fibrosis exacerbates and eventually turns into HFrEF. Patients will have prominent exercise intolerance (12). The change in myocardial microcirculation is noticeable now. Severe collagen deposition results in coronary arteriolar sclerosis, basement membrane thickening, and capillary microaneurysm (20). Cardiac remodeling exceeds myocardial repair capacity. LV mass, volume, and wall thickness continue to increase (12, 15), accompanied by LV dilatation. At this time, focal fibrosis can be detected by positron emission tomography (PET), computed tomography (CT), and magnetic resonance imaging (MRI) (16). Based on diastolic dysfunction, the signs of systolic dysfunction begin to appear, manifested as shortened ejection period, prolonged performance before ejection, and increased filling pressure. Ischemic heart disease and severe CAN are the two major complications at this stage (17–20). At the subcellular level, increased ROS and inflammation (13), contractile and regulatory protein expression abnormalities, and impaired myocardial insulin signal transduction lead to decreased endothelial nitric oxide synthase activation and bioavailable nitric oxide levels (42).

Even if this trisection model seems perfect, some believe that LVH is the first performance of the first stage rather than in the middle (15). Because at the cellular level, the activation of neurohumoral mechanisms such as hyperglycemia, insulin resistance, renin-angiotensin-aldosterone system (RAAS), and sympathetic nervous system (SNS) lead to cardiomyocyte hypertrophy, stiffness, and fibrosis, which is sufficient to trigger this process. Meanwhile, free fatty acid (FFA) accumulation, calcium homeostasis imbalance, and GLUT-1 and -4 depletion contribute to myocyte injury at the molecular level (15).

Furthermore, some pathological mechanisms like microvascular perfusion damage remain debatable (16). The microcirculation disturbance is recognized in the late stage of this disease, but it is not clear in the early stage. The current known decrease in myocardial blood flow reserve is most likely related. DCM is undoubtedly a DM microcirculation complication if microcirculation disturbance runs through the process.

#### Four-division

Some scholars divide DCM into four stages with a clear hierarchy based on NYHA classification and AHA staging (43–46). They emphasize that DCM is the earliest contributor to HF in patients with DM in an ideal state. In this model, the presence or absence of comorbidities is regarded as the watershed of HF. The early stages (stages A and B) are characterized by simple DCM, mainly caused by the pathophysiology of diabetes. The latter stages (stages C and D) show that DCM coexists with other complications, and diabetes is a secondary factor. This scheme extends the traditional definition of diabetic cardiomyopathy, considering the pathophysiological characteristics, echocardiography changes, and periodic changes in serum biomarkers. The stages are:

Stage A (diastolic dysfunction): no clinical symptoms. Patients usually have normal cardiac structure and function, at most subclinical cardiac structure and function abnormalities, normal ejection fraction (LVEF), and no non-DCM complications. However, patients often show hypertrophic and restrictive phenotypes because LVH increases ventricular stiffness, leading to diastolic dysfunction. This is the earliest stage of DCM and can be detected in 28% to 75% of asymptomatic diabetic patients (14). Finally, 36.9% of patients with stage A will progress to symptomatic heart failure (47).

Stage B (systolic and diastolic dysfunction): mild/moderate physical activity limitation. Structural and functional abnormalities appear in the heart, but no non-DCM complication exists. In addition to diastolic dysfunction, patients may have decreased ventricular dilatation and ejection fraction. Secondary complications such as coronary heart disease and hypertension are possible but are not severe.

Stage C (mild complications): severe physical activity limitation. Cardiac dysfunction and LVEF decline worsen. Non-DCM complications, such as hypertension, microvascular disease, and viral heart disease, begin to appear. Patients may have myocarditis and coronary atherosclerosis but no coronary heart disease (14).

Stage D (severe complications): there are still symptoms or imminent death at rest. Biventricular refractory heart failure lasts. Non-DCM complications are severe, including dilatation, fibrosis, microvascular and macrovascular lesions, and obvious native coronary heart disease. It can also be combined with myocardial overload after myocardial infarction, with or without hypertensive heart disease.

In addition, there are different beliefs. Because DM is a clear risk factor for HF, some researchers believe it enters phase A as long as it occurs. Further progress is manifested as simple diastolic dysfunction; this is a split in phase A of the above program, but this model does not refer to complications. The specific stages can be summarized as follows: stage A (risk factor stage): simple diabetes; stage B (HFpEF): left ventricular diastolic dysfunction (LVDD) without symptoms but with LVH; phase C (HFrEF): EF decreased with obvious symptoms and signs such as dyspnea and pulmonary congestion; stage D (terminal stage): heart failure that is difficult to treat (48).

#### Six-division

After nearly 30 years of accumulation of CMR-derived myocardial mechanics data, according to the order of structure and dysfunction, a view of the DCM six points method was created (49).

In the first stage, myocardial interstitial fibrosis occurs; this is detected in a diagnostic test. Researchers used sensitive methods to measure the left ventricle’s multidirectional strain and strain rate. The overall longitudinal, circumferential, and radial strains were not significantly different compared to the healthy control group, but ECV (extracellular volume, representing interstitial fibrosis) in T2D patients was significantly high (50). AGEs accumulation or myocardial interstitial neovascularization may cause ECV dilatation. An animal study also showed that ECV first increased after three months of diabetes induction. The ultrasonic-derived LV radial strain rate changed after six months, and radial strain damage was observed after nine months (51). These studies have confirmed that extracellular fibrosis develops first, and mechanical function impairs. The early stage of interstitial fibrosis may not be sufficient to cause mechanical damage to the left ventricle, which is the reason for independently dividing it into stages.

The second stage of diabetic cardiomyopathy is LV mechanical dysfunction. Echocardiography and nuclear magnetic resonance studies have shown that in the systolic and early diastolic stages (52), the multidirectional strain of myocardial layers in diabetic patients decreased significantly (53–56), and a few strains increased compensatorily (53). Liu et al. (57) confirmed the decrease of diastolic longitudinal and circumferential strain rates in patients with T2D. In contrast, longitudinal strain and peak systolic longitudinal strain rates decreased in patients with T2D over 5 years (57).

The third stage is LVDD. Nuclear magnetic resonance (30, 58) studies have shown diastolic dysfunction in pre-diabetes (59), type 1 diabetes (T1D) (60, 61), and T2D (55) patients (children (62) and adults (63)). Moreover, from this stage on, changes can be detected by conventional echocardiography (55, 59, 60).

The fourth stage is LVH, the fifth stage is HFpEF, and the final stage is HFrEF. Not all diabetic patients will progress through all stages.

### Double clinical phenotypes

One thing in common in the above views regardless of the number of DCM phases: admitting that DCM has only one phenotype. However, some scholars believe that DCM has two phenotypes. Furthermore, these two phenotypes are not continuous stages of DCM but independently evolved into heart failure with LVEF preservation or reduction (64).

Researchers have found the phenotype-specific mechanisms of HFpEF and HFrEF, namely coronary microvascular endothelial dysfunction in HFpEF and myocardial cell death in HFrEF. The involvement priority of endothelial cell or cardiomyocyte cell compartments determines the development direction of DCM. In obese patients with T2D, abnormal glucose, lipid metabolism, and insulin resistance coexist and tend to occur in DCM’s restrictive/HFpEF phenotype. T1D patients with autoimmune tendency are more likely to develop into dilated/HFrEF phenotype.

### A hypothesis: subclinical hyperfunction

Early DCM was not well understood. It was generally believed before that the cardiac function of patients with DCM remained normal initially. However, a new hypothesis is that DCM has a stage of myocardial systolic hyperfunction (65). This myocardial hyperdynamic state is short-lived and is the onset of the asymptomatic subclinical stage.

Based on the findings of a clinical study, this hypothesis adds to the early stage of DCM evolution. Hensel KO et al. used speckle tracking echocardiography to study the short course (4.3 ± 3.5 years) of type 1 diabetic adolescents without complications (62). Compared to the healthy control group, patients with type 1 diabetes showed overall longitudinal and circumferential LV myocardial systolic ability enhancement under rest and load. However, this situation is more evident in patients with a longer disease course, indicating that a high dynamic state may continue for several years.

Some evidence has been found in human and animal models. M-mode and Doppler ultrasound studies found that LV systolic ability increased in children with simple diabetes (66, 67). This phenomenon occurred only in normal albuminuria. When microalbuminuria occurred, LV systolic ability returned to normal (68). Studies on MRI also found that young adults with T1D showed signs of increased LV torsion at the early stage (69, 70). Few studies have revealed its pathophysiological mechanism. Only one animal model study suggests that it may be associated with increased plasma volume and sympathetic activation (27).

In short, this high LV systolic capacity is more like compensation for reduced myocardial efficiency. However, this is not widely reported because myocardial hypercontraction has no clinical symptom and can only be detected using sensitive methods. Therefore, more clinical data are required to confirm this hypothesis.

## Overall clinical findings

Many studies have reported that DCM does not have any obvious clinical manifestations. It has nonspecific symptoms and signs of heart failure only when it progresses to the advanced stage. Therefore, auxiliary examination methods are the only ways to diagnose DCM. Previously, it was difficult to diagnose DCM because noninvasive techniques were inaccurate. Restrictive phenotype determination still required cardiac catheterization, while dilated phenotype required myocardial biopsy (48, 71); this made it difficult for humans to uncover the mysterious veil of DCM.

Noninvasive cardiac imaging has come a long way in the last 40 years, and because of its safety, simplicity, and accuracy, it is becoming increasingly important in diagnosing DCM. Nowadays, people can characterize DCM in metabolism, structure, and function. Numerous diagnostic clinical findings have been accumulated. They are summarized and listed below (**Tables 1**–**3**). All cardiac indicators differ meaningfully between diabetic patients and healthy people are included. Because DCM’s clinical phenotypes and mechanisms are different in T1D and T2D, we sorted them out according to the type (64, 166). Given that the mixed population study (including T1D and T2D) still has a certain reference value, we summarized these studies together because it represents the general rule of DM.

**Table 1:** Main clinical findings of biomarkers for cardiac dysfunction in T1D and T2D patients.

Biomarkers		T1D	T2D	DM (T1D+T2D)
Conventional indicators	NT-proBNP	↑ (72)	↑ (73)	↑ (74)
	hs-cTnT, BNP, NLR		↑ (75–78)	
	TNF-α, IL-6, AGEs, Creatinine		↑ (79)	
	GDF-15, Galectin-3, MMP-7, PIP, CT-1		↑ (80–84)	
MiRNA	mir-223		↑ (85)	
	mir-21		↓ (86)	
LncRNA	lncRNA NKILA			↑ (87)

NLR, Neutrophil to lymphocyte ratio.

↑ means the figure is higher than that of healthy people, while ↓ means the figure is lower than that of healthy people.

**Table 2:** Main clinical findings of ultrasound for cardiac dysfunction in T1D and T2D patients.

Instrumental examination					T1D	T2D	DM (T1D+T2D)
Conventional Doppler Echocardiography	Epicardium	Structure	EFT		↑ (88, 89)	↑ (90)	↑ (91)
	LA	Structure	LAV index			↑ (55, 92, 93)	
		Function	LAEF			↓ (94, 95)	
			LAEV			↓ (94)	
			atrial filling fraction				↑ (96, 97)
	LV	Structure	interventricular septum thickness			↑ (55)	
			posterior wall thickness			↑ (55)	↑ (97)
			relative wall thickness			↑ (55, 92, 94)	
			LV mass index		↑ (98)	↑ (55, 92, 94)	
			LVEDV		↓ (99)		
		Function	EDT		↑ (61, 100, 101)	↑ (55, 92, 102, 103)	↑ (104)
						↓ (93)	
			E/A		↓ (61–63, 100, 105)	↓ (55, 83, 92–94, 102, 103, 106)	↓ (97, 104, 107)
					↑ (108)		
			IVRT		↑ (99–101, 105)	↑ (92, 102, 103)	↑ (97)
						↓ (93)	
			LVEF			↓ (92)	
			fractional shortening			↓ (92)	
	RV	Function	IVRT		↑ (61)		
			MPI		↑ (61)		
			E/A		↓ (63, 109)	↓ (83)	
TDI	LA	Function	strain			↓ (95)	
	LV	Function	E/e′			↑ (55, 93, 110, 111)	↑ (104)
						↓ (72, 92)	
			MPI			↓ (92, 112)	
			interventricular septum	systolic strain			↓ (54)
				SRs			↓ (54)
				SRe			↓ (54)
			lateral wall	systolic strain			↓ (54)
				SRs			↓ (54)
				SRe			↓ (54)
			GRS			↓ (113)	
			longitudinal strain	global	↓ (108, 114)	↓ (113)	
				endocardia		↓ (113)	
				mid-myocardial		↓ (113)	
				epicardial		↓ (113)	
			circumferential strain	global		↓ (113)	
				endocardia		↓ (113)	
				mid-myocardial		↓ (113)	
				epicardial		↓ (113)	
			LV twist			↑ (113)	
	RV	Function	basal segment	systolic strain			↓ (54)
				SRs			↓ (54)
				SRe			↓ (54)
			apical segment	systolic strain			↓ (54)
				SRs			↓ (54)
				SRe			↓ (54)
			GLS		↓ (109)		
			FWLS		↓ (108)		
2D STE	LA	Structure	LAV index				↑ (115)
		Function	strain	global			↓ (115)
				reservoir	↓ (116)		
				conduit	↓ (116)		
			LA stiffness		↑ (116)		
	LV	Function	GWI			↓ (117)	
			GWE			↓ (117)	
			GWW			↑ (117)	
			longitudinal strain	global	↓ (118)	↓ (92, 110, 117, 119)	↓ (120)
				endocardial	↓ (121)	↓ (55)	
				mid-myocardial	↓ (121)	↓ (55)	
				epicardial	↓ (121)	↓ (55)	
			circumferential strain	global	↓ (118)		
				endocardial		↓ (55)	
				mid-myocardial	↓ (121)	↓ (55)	
				epicardial	↓ (121)	↓ (55)	
			radial strain	basal	↓ (118)		
				subendocardial		↓ (119)	
	RV	Function	free wall strain			↓ (122)	
			GLS		↓ (118)		
3D E	LA	Structure	LAV index			↑ (123)	
		Function	LAEF			↓ (123)	
			GLS			↓ (124)	
			GCS			↓ (124)	
			GAS			↓ (124)	
3D STE	LV	Function	GLS		↓ (125)		↓ (126)
			GCS				↓ (126)
			GRS				↓ (126)
			GAS				↓ (126)
Physical SE	LV	Function	GLSR at rest and during exercise		↑ (62)		
			GCSR at rest		↑ (62)		
			longitudinal reserve index	diastolic		↓ (127)	
				systolic		↓ (127)	
Pharmacological SE	LV	Function	rotation	apical		↓ (128)	
				basal		↓ (128)	
			LV twist			↓ (128)	
			ΔLVEF			↓ (129)	
Stress Doppler wire	Coronary Artery	Structure	coronary diameter			↓ (129)	
		Function	maximal hyperemic flow			↓ (129)	
			coronary flow velocity			↑ (129)	
			CFR			↓ (129)	

Conventional doppler echocardiography contains 2D, M-mode, color, pulsed, and continuous-wave doppler echocardiography. Doppler echographies based on tissue doppler imaging are included in "TDI", for example, SRI. Physical stress echocardiography contains tissue doppler echocardiography and speckle tracking echocardiography. Pharmacological stress echocardiography contains conventional echocardiography and speckle tracking echocardiography.

CFR, coronary flow reserve; EDT, E deceleration time; EFT, Epicardial fat thickness; E/A, ratio of the early to the late peak diastolic transmitral flow velocity; FWLS, free wall longitudinal strain; GRS, global radial strain; GLS, global longitudinal strain; GCS, global circumferential strain; GWI, global myocardial work index; GWW, global wasted work; GWE, global work efficiency; GAS, global area strain; GLSR, global longitudinal strain rate; GCSR, global circumferential strain rate; IVRT, isovolumic relaxation time; LV, left ventricular; RV, right ventricular; LAV, left atrial volume; LVEF, left ventricular ejection fraction; LA, left atrium; LAEF, left atrial ejection fraction; MPI, myocardial performance index; RA, right atrium; SRs, peak systolic strain rate; SRe, peak early diastolic strain rate; SE, stress echocardiography; TDI, tissue doppler imaging; 2D STE, two-dimensional speckle tracking echocardiography; 3D E, three-dimensional echocardiography; 3D STE, three-dimensional speckle tracking echocardiography.

↑ means the figure is higher than that of healthy people, while ↓ means the figure is lower than that of healthy people.

**Table 3:** Main clinical findings of CT/MRI/PET and other methods for cardiac dysfunction in T1D and T2D patients.

Instrumental examination						Pre-DM	T1D	T2D	DM (T1D+T2D)
ECG	QTc max. interval							↑ (130)	
	QTc dispersion							↑ (130)	
	T peak-Tend dispersion							↑ (130)	
	QRS-T angle							↑ (131)	
CT	LV mass						↓ (132)		
	LV volume						↓ (132)		
	RV volume						↓ (132)		
MRI	LA	Function		GCS				↓ (133)	
				GRS				↓ (133)	
				GLS				↓ (133)	
				LA ejection fraction	total			↓ (134, 135)	
					passive			↓ (135)	
	LV	Structure		LV mass				↑ (5)	↑ (136)
				LV mass index				↑ (5, 137, 138)	
				LVEDV				↓ (135)	
				LVEDV index				↓ (134, 139)	
				LVESV index				↓ (139)	
				LV mass/volume				↑ (5, 134, 135, 137, 139)	
				LV wall thickness		↑ (140)		↑ (138, 140)	
		Function		diastolic strain rate	inferior septal		↓ (141)		
				free wall region		↓ (141)		
				diastolic relaxation fraction			↑ (141)		
				systolic stretch fraction			↑ (141)		
				apical FD				↑ (137)	
				torsion angle					↑ (136)
				E peak	acceleration			↓ (30)	
				deceleration			↓ (30)	
				filling rate			↓ (30)	
				E/A			↓ (30)	
				peak strain	longitudinal			↓ (142)	
				circumferential			↓ (142)	
				peak diastolic strain rate	longitudinal			↓ (57, 137, 142)	
				circumferential			↓ (5, 57, 142)	
				radial			↓ (57, 142)	
	RV	Structure		RVEDV				↓ (143)	
				RVESV				↓ (143)	
		Function		GLS				↓ (133)	
				stroke volume				↓ (143)	
				pulmonary flow acceleration time				↑ (143)	
				PERPV				↓ (143)	
				PFRE				↓ (30, 143)	
				PDGE				↓ (30, 143)	
	Global	Metabolism		Lactate				↑ (144)	
				Bicarbonate				↓ (144)	
				Bicarbonate/Lactate				↓ (144)	
		myocardial perfusion		upslope				↓ (57, 145)	
				max signal intensity				↓ (57, 145)	
				time to maximum signal intensity				↑ (57, 145)	
				BOLD SID				↓ (146)	
				MPR			↓ (70)	↓ (146, 147)	↓ (136)
MRI T_1_-mapping	Structure		LV mass/volume			↑ (148)		↑ (145, 147)	↑ (148)
			native T_1_ values				↑ (149)		
			ECV			↓ (148)	↑ (149)	↑ (150, 151)	↓ (148)
			cell volume			↑ (148)			↑ (148)
	Function	myocardial function		myocardial T_1_ time					↓ (152)
				LVEDV				↓ (138, 147)	
				LVEDV index				↓ (147)	
				systolic strain				↓ (138)	
				systolic torsion			↑ (149)		
				systolic torsion rate			↑ (149)		
				systolic E_cl_			↑ (149)		
				rates of systolic E_cl_			↑ (149)		
				rates of diastolic E_cl_			↑ (149)		
				LV stroke volume				↓ (147)	
				LV stroke volume index				↓ (147)	
		Microvascular function		∆T_1_				↓ (147)	
^1^H-MRS	Myocardial lipid content							↑ (5, 144, 153)	
^31^P-MRS	PCr/ATP							↓ (5, 30, 144, 146, 154)	
G-Spect	phase bandwidth							↑ (155)	
PET	myocardial glucose metabolism		uptake				↓ (156)	↓ (157–159)	↓ (160)
			uptake/plasma insulin					↓ (161)	
			utilization				↓ (162)	↓ (157)	
			utilization rate/insulin				↓ (163)		
			oxidation				↓ (156)	↓ (157)	
			glycogen deposition					↓ (157)	
			glycolysis				↓ (156)	↓ (157)	
			glycogen synthesis				↓ (156)		
	myocardial fatty acid metabolism		uptake					↑ (159)	
			utilization				↑ (162)	↑ (158, 161)	
			oxidation				↑ (162)	↑ (158, 161)	
			percent oxidation				↑ (162)	↓ (161)	
			esterification					↑ (161)	
	myocardial oxygen consumption		MVO_2_				↑ (162, 163)		
	myocardial perfusion		ΔCBF					↓ (164)	
Cardiopulmonary Exercise Testing	peak oxygen uptake							↓ (55)	
	peak oxygen consumption							↓ (95)	
	oxygen pulse							↓ (55)	
	ventilation/carbon dioxide slope							↑ (55, 95)	
Coronary Angiography	microvascular function		CFR					↓ (165)	
			MRR					↓ (165)	

apical FD, apical fractal dimension of trabeculations; BOLD SID, blood-oxygen level-dependent signal intensity change; CT, computed tomography; CBF, coronary blood flow; CFR, coronary flow reserve; EDV, end-diastolic volume; ESV, end-systolic volume; E/A, ratio of the early to the late peak diastolic transmitral flow velocity; ECG, electrocardiograph; ECV, extracellular volume; E_cl_, the shear strain component associated with twist; GCS, global circumferential strain; GLS, global longitudinal strain; GRS, global radial strain; LA, left atrium; LV, left ventricle; LVMI, left ventricular mass index; MRI, magnetic resonance imaging; MRS, nuclear magnetic resonance spectrum; MPR, myocardial perfusion reserve; MVR, ratio of left ventricular mass at end diastole to end-diastolic volume; MRR, microvascular resistance reserve; MVO_2_, myocardial oxygen consumption; PET, positron emission tomography; PSDR, peak diastolic strain rate; PERPV, peak ejection rate across the pulmonary valve; PFRE, peak filling rate of the early filling phase; PDGE, peak deceleration gradient of the early filling phase; RA, right atrium; RV, right ventricle.

↑ means the figure is higher than that of healthy people, while ↓ means the figure is lower than that of healthy people.

Serological markers, echocardiography, cardiac magnetic resonance imaging, and positron emission tomography are widely used as the main tools to explore the changes in DCM heart. Because CMR is readily unavailable and expensive and has contraindications, and may not be suitable for patients with autism or metal implants, the application of CMR remains in the field of scientific research. Although echocardiography has inter-observer variability, and the assessment of right ventricular structure and function is often more nonstandard in the heart’s four chambers, it is generally the preferred method for diagnosing DCM and tracking disease progression (48). Adding a series of new serological markers makes it more possible to implement a large screening because this is the simplest method. Many plasma/serum substances reflect changes in myocardial metabolism, structure, and function and may indicate prognosis (167). Metabolic changes in DCM can be detected using emerging magnetic resonance spectroscopy imaging, which appears to be the earliest detectable change in DCM. We also included DCM heart changes found by invasive techniques (**Tables 2**, **3**), hoping to present a full spectrum of DCM lesions.

## Screening and diagnosis criteria

### Screening criterion

Diabetic cardiomyopathy screening knowledge is still insufficient to build up a criterion, but some useful information has emerged. Asymptomatic patients with T1D or T2D are considered to need further examination when they have risk factors as follows (168) (1): Longstanding DM (2); Poorly controlled DM (3); Microvascular complications of DM: diabetic kidney disease (↑UACR, ↓eGFR), retinopathy, and neuropathy. It is thought to be more suspicious if evidence of cardiac LV dysfunction or LVH as well as exercise intolerance can be found by doppler ultrasound or cardiopulmonary exercise test (168, 169). Additionally, five serological or urinary markers may be currently available for screening. Earlier researches have shown that, while BNP is a more visible indicator of diastolic dysfunction than hs-CRP, its sensitivity and specificity are low (170, 171). However, with the advancement of research, increasing evidence has confirmed that BNP or NT-proBNP is closely related to heart failure and is still recommended as an early screening for DCM (172, 173). Two prospective randomized controlled trials have confirmed that a new standard (BNP≥50 pg/mL or NT-proBNP>125 pg/ml) can screen high-risk DM populations properly to reduce the incidence of cardiovascular hospitalizations/death or heart failure (173, 174). A study has also shown that NT-proBNP level below 125 pg/mL helps role out the possibility of asymptomatic LV dysfunction in the DM population (73). What’s more, HbA_1_c levels are proportional to the degree and frequency of diastolic dysfunction (175). Interestingly, testing microalbuminuria in people with diabetes helps identify their risk of diastolic dysfunction (78). However, the detection results of these indicators cannot directly confirm myocardial changes, so they must be determined by imaging tests.

### Diagnosis criterion

As mentioned above, diabetic cardiomyopathy shows metabolic, structural, and functional changes. Therefore, many scholars are trying to find a cut-off point to establish the diagnosis of DCM. A study published in 2013 provided initial diagnosis clues of DCM (176). After other heart diseases are ruled out, changes as follows should be evaluated: The structural changes include (1) LV hypertrophy assessed by 2D echocardiography or CMR (2); Increased integrated backscatter in the LV (septal and posterior wall); and (3) Late Gd-enhancement of the myocardium in CMR. The functional changes include (1) LVDD assessed by pulsed Doppler echocardiography and TDI (2); LV systolic dysfunction (LVSD) demonstrated by TDI/SRI; and (3) Limited systolic and/or diastolic functional reserve assessed by exercise TDI. The metabolic changes include (1) Reduced cardiac PCr/ATP detected by ^31^P-MRS; (2) Elevated myocardial triglyceride content detected by ^1^H-MRS.

In 2015, researchers proposed different phenotypes of DCM development and their diagnostic criteria (64). The phenotypes should both meet the following conditions first: (1) Presence of DM; (2) Exclusion of CAD, valvular, or congenital heart disease; and (3) Exclusion of hypertensive heart disease (=DBP<90 mmHg). Based on this, the diagnostic criterion of Dilated/HFrEF Phenotype should include (1) Exclusion of viral myocarditis by endomyocardial biopsy; (2) LVEF<50%, LVEDVI>97 mL/m^2^. While the criterion of Restrictive/HFpEF Phenotype should include: (1) Exclusion of infiltrative heart disease by endomyocardial biopsy; (2) LVEF>50%, LVEDVI<97 mL/m^2^; and (3) E/e’ >15 or 8<E/e’ <15+LAVI>40 mL/m^2^ or 8<E/e’ <15+BNP>200 pg/mL or 8<E/e’ <15+atrial fibrillation or 8<E/e’<15+LVH (LVMI♀>122 g/m^2^; LVMI♂>149 g/m^2^).

In 2021, a study (168) put forward a new diagnostic criterion based on the four-division method. Acknowledging only one phenotype exists, they believe stage B is the best time to diagnose DCM. Patients should have at least one of the following echocardiographic abnormalities: (1) LVH, defined as LV mass index (LVMi) > 115 g/m^2^ in men and > 95 g/m^2^ in women; (2) LAE, defined as left atrial volume index (LAVi) ≥ 34 mL/m^2^; (3) abnormal ratio of mitral inflow peak early diastolic velocity (E) to tissue Doppler mitral annular early diastolic velocity (e’), defined as E/e’ ≥ 13; (4) impaired GLS, defined as GLS < 18%.

## Clinical practice

Currently, the treatment for DCM is mainly divided into three kinds: conventional cardiovascular drugs, anti-glycemic drugs, and new therapies such as CoQ10, MicroRNA and Stem cell therapy (14). Many reviews have focused on the outcomes of the trials of these therapies (14, 168, 177). Although the number of the trials is large, most of them recruited mixed population, resulting in invalid data for judging whether it is effective for treating DCM. What’s worse, each kind has certain limitations now. For example, conventional cardiovascular drugs only apply when DCM develops into more obvious cardiac symptoms. Conventional anti-glycemic drugs have an insignificant benefit, and only SGLT2i (a new kind of anti-glycemic drug) is recommended as the first-line medicine for DCM. Moreover, new therapies still have defects such as clinical trial failure and insignificant safety. Therefore, more studies must be conducted to find suitable drugs. The following table (**Table 4**) summarizes the randomized controlled trials (RCTs) related to DCM in the last 10 years, hoping to inspire clinical drug use.

**Table 4:** Main RCTs on diabetic cardiomyopathy.

Author (Year)	Target	Drug	Queue size	Outcomes	Refs
Hiroki Oe, Kazufumi Nakamura, Hajime Kihara (2015)	DPP4	Sitagliptin VS Voglibose	100	Sitagliptin reduced HbA_1_c levels more greatly than voglibose does, but neither was associated with improvement in the echocardiographic parameters of LV diastolic function in patients with diabetes.	(178)
Hirotsugu Yamada, Atsushi Tanaka, Kenya Kusunose (2017)	DPP4	Sitagliptin	115	Adding sitagliptin to conventional antidiabetic regimens for 24 months in T2D patients attenuated the exacerbation in E/e′, the echocardiographic parameter of diastolic dysfunction.	(179)
Alexander J.M. Brown, Chim Lang, Rory McCrimmon (2017)	SGLT-2	Dapagliflozin	66	Dapagliflozin treatment significantly reduced left ventricular mass accompanied by reductions in systolic BP, body weight, visceral and subcutaneous adipose tissue, insulin resistance, and hs-CRP.	(180, 181)
Daisuke Matsutani, Masaya Sakamoto, Yosuke Kayama (2018)	SGLT-2	Canagliflozin	37	Canagliflozin could improve left ventricular diastolic function within 3 months in patients with T2DM, which was especially apparent in patients with substantially improved hemoglobin values.	(182)
Satoshi Oka, Takahiko Kai, Katsuomi Hoshino (2021)	SGLT-2	Empagliflozin	35	The positive effects of empagliflozin on LV dysfunction were more remarkable in early than in advanced DCM.	(183)
Sharmaine Thirunavukarasu, Nicholas Jex, Amrit Chowdhary (2021)	SGLT-2	Empagliflozin	18	Empagliflozin ameliorated the cardiac energy metabolism, regressed adverse myocardial cellular remodeling, and improves cardiac function.	(184)
Rebecca L. Scalzo, Kerrie L. Moreau, Cemal Ozemek (2017)	GLP1R	Exenatide	23	Administrating exenatide improved cardiac function and reduced arterial stiffness; however, these changes were not accompanied by improved exercise capacity.	(185)
Weena J. Y. Chen, Michaela Diamant, Karin de Boer (2017)	GLP1R/IR	Exenatide VS Insulin Glargine	33	Exenatide or insulin glargine had no effects on cardiac function, perfusion or oxidative metabolism.	(186)
Vaia Lambadiari, George Pavlidis, Foteini Kousathana (2018)	GLP1R/AMPK	Liraglutide VS Metformin	60	Six-month treatment with liraglutide improved arterial stiffness, LV myocardial strain, LV twisting and untwisting and NT-proBNP in subjects with newly diagnosed T2D, which is related to anti-oxidative stress.	(187)
Maurice B. Bizino, Ingrid M. Jazet, Jos J. M. Westenberg (2019)	GLP1R	Liraglutide	49	Liraglutide reduced early LV diastolic filling and LV filling pressure, thereby unloading the left ventricle. LV systolic function reduced and remained within normal range.	(188)
Elisabeth H.M. Paiman, Huub J. van Eyk, Minke M.A. van Aalst (2020)	GLP1R	Liraglutide	47	Liraglutide did not affect LV diastolic and systolic function, aortic stiffness, myocardial triglyceride content, or extracellular volume in Dutch South Asian type 2 diabetes patients with or without coronary artery disease.	(189)
Ignatios Ikonomidis, George Pavlidis, John Thymis (2020)	GLP-1R/ SGLT-2	Liraglutide + Empagliflozin	160	Treatment with GLP-1RA, SGLT-2i, and their combination within 12 months showed a greater improvement of vascular markers and effective cardiac work than insulin treatment in T2DM. The combined therapy was superior to either insulin or GLP- 1RA and SGLT-2i separately.	(190)
Elisa Giannetta, Andrea M. Isidori, Nicola Galea (2012)	PDE5	Sildenafil	54	Selective phosphodiesterase type 5 inhibitor, sildenafil, acts directly on the myocardium through its anti-remodeling effect to ameliorate LV concentric hypertrophy associated with altered myocardial contractility dynamics in the early stages of diabetic cardiomyopathy.	(191)
S. Giannattasio, C. Corinaldesi, M. Colletti (2019)	PDE5	Sildenafil	46	Sildenafil can control DCM progression through IL-8 targeting at the systemic and cellular levels.	(192)
Peng Zhao, Jie Zhang, Xian-Gang Yin (2013)	3-KAT	Trimetazidine	80	Trimetazidine treatment can improve cardiac function and physical tolerance and decrease the inflammatory response.	(193)

AMPK, Adenosine 5’-monophosphate-activated protein kinase; DPP4, Dipeptidyl-Peptidase 4; GLP1R, glucagon-like peptide 1 receptor; IR, insulin receptor; PDE5, phosphodiesterase type 5; SGLT-2, sodium-dependent glucose transporters 2; 3-KAT, long-chain 3-ketoacyl coenzyme A thiolase.

## Discussion

### Phase of progression

Although the progression phase contains some theoretical components, it stems from our understanding of the entire disease process, which has important guiding significance for clinical practice and scientific research. The differences among them are mainly reflected in three points: the phenotypes of the disease, the beginning of the disease, and the stages of the disease. Different phenotypes require different treatments, which is relevant to developing guidelines. If DCM only has one phenotype, it is necessary to consider the secondary prevention of HFpEF progression to HFrEF, and appropriate animal models should also be considered in basic research. If DCM exhibits two distinct phenotypes, clinical trials and basic research must be divided into sub-fields to be further investigated. It is also important to define the beginning of the disease. Although many researchers agree that the myocardial injury caused by diabetes begins at the onset of diabetes, some scholars have pointed out through epidemiological analysis that the natural history of diabetic cardiomyopathy has begun as early as the metabolic syndrome period. Patients who have elevated inflammatory markers and microalbuminuria are at risk of developing heart failure (14). Therefore, clarifying the starting point of DCM is of great value for formulating disease screening strategies, and early treatment can delay the progress of DCM. Furthermore, the division of disease stages, which provides strong support for clinicians in evaluating the severity of DCM patients and selecting the intervention time, should not be ignored. Moreover, it also affects the evaluation of drug efficacy because drugs have different benefits for different stages.

We have summarized all the proposed DCM Spatio-temporal evolution models (**Table 5**). The dichotomy is the most concise model for clinicians to master and can be easily popularized in communities. However, it should be noted that structural and metabolic changes that go unnoticed are the culprits of dysfunction. The trisection model does not advance much more than the dichotomy but emphasizes the discreteness, concealment, and progressive fibrosis of early dysfunction. Unless in medical institutions with advanced technological means, the clinical practice remains a dichotomy because early changes cannot be detected promptly.

**Table 5:** Summary of Phases of Progression.

		Stages	Cellular mechanisms	Tissue and organ changes	Metabolism changes	Functional changes	Structural changes	Common complications	Means of auxiliary examination
Single clinical phenotype	Dichotomy	Early stage	Hyperglycemia, insulin resistance, impaired Ca^2+^ handling, hyperactivation of the SNS and RAAS, and inflammation	/	Increased FFA and AGE deposition	Diastolic dysfunction, increase in left atrial filling pressure, and elevated LV end-diastolic pressure	LV concentric hypertrophy, increased ventricular stiffness, and left atrial enlargement	/	/
		Advanced stage	Microvascular endothelial dysfunction, neurohormonal impairment, inflammation, impaired Ca^2+^ handling	Cardiac fibrosis and cardiomyocyte apoptosis	/	Diastolic dysfunction, systolic dysfunction	LV dilation, cardiac remodeling, and LV hypertrophy	/	/
	Trichotomy	Early stage	Depletion of GLUT-1 and -4, Ca^2+^ homeostasis changes, hyperglycemia, insulin resistance, endothelial dysfunction, activated RAAS and SNS, oxidative stress, and inflammation	Very small pathophysiological changes in myocytes, such as cardiomyocyte substructural changes	Increased FFA and Carnitine deficiency	Prolonged isovolumetric relaxation, increased atrial filling, impaired early diastolic filling, increased cardiac relaxation and stiffness	Decreased myocardial blood flow reserve	/	Sensitive methods such as strain, strain rate, and myocardial tissue velocity, MRI, CTA, and IVUS
		Middle stage	Defects in Ca^2+^ transport, hormone disorders (increased AT II and TGF-β1, reduced IGF-I), activated RAAS, accumulation of AGEs, collagen deposition	Cardiomyocyte loss and fibrosis, high cardiomyocyte resting tone	/	Diastolic dysfunction, slightly impaired ejection function, increased left ventricular stiffness	Left ventricular hypertrophy, slightly increased LV mass, wall thickness or size	Mild CAN	Conventional echocardiography, sensitive methods (the same as techniques above)
		Late stage	Myofibril reduction, contractile and regulatory protein expression abnormalities, increased formation and deposition of collagen	Advanced cardiac fibrosis, cardiomyocyte necrosis, microvascular changes and spasm	/	Significant deterioration of coronary microcirculation and systolic and diastolic function	Significantly increased LV size, wall thickness and mass	Hypertension, ischemic Heart Disease, Severe CAN	Conventional echocardiography
	Four-division	Stage A (diastolic dysfunction)	Altered Ca^2+^ homeostasis, depletion of GLUT-1 and -4, increased ROS and inflammation, disregulated miRNA and exosomes	Steatosis	/	Diastolic dysfunction with normal EF, mild decrease in systolic strain of both atria and ventricles	Increased LV mass, decreased tissue velocities	/	LGE ,^1^H-Spectroscopy, and ^31^P-Spectroscopy
		Stage B (systolic and diastolic dysfunction)	Stage A mechanisms + Acticated RAAS, cytokine damage (reduced IGF-I and increased TGF-β1), hyperglycemia, AGE formation, insulin resistance	Apoptosis, necrosis, and fibrosis	loss of cardiac metabolic flexibility	Combined systolic and diastolic dysfunction, right and left atrial and ventricular involvement	Increased LV wall thickness and mild cavity dilatation	Mild CAN	LGE, ^1^H-Spectroscopy, and ^31^P-Spectroscopy
		Stage C (mild complications)	Stage A and Stage B mechanisms + microvascular changes	Apoptosis, necrosis, and fibrosis	Overt DM	Diastolic dysfunction, systolic dysfunction, decreased LVEF, and pulmonary hypertension	Concentric hypertrophy or indeterminate hypertrophy (magnification) or eccentric hypertrophy, cavity dilatation, Abnormal EMB	Microvascular disease/coronary atherosclerosis without obstructive CHD, HTA, severe CAN	LGE, ^1^H-Spectroscopy, and ^31^P-Spectroscopy
		Stage D (severe complications)	the same as Stage C mechanisms, but more severe	Apoptosis, necrosis, and fibrosis	Overt DM	Moderate-severe systolic dysfunction and biventricular refractory heart failure	Eccentric hypertrophy	Overt ischemia/infarct causing HF, micro- and macroangiopathic complications (e.g. CHD and CKD), severe CAN	LGE, ^1^H-Spectroscopy, and ^31^P-Spectroscopy
	Six-division	/	Hyperglycemia, insulin resistance, changed Ca^2+^ homeostasis, increased ROS and inflammation, mitochondrial damage, cytokine damage, inactive iNOs, endothelial cell dysfunction, activated SNS and RAAS (before I stage)	Interstitial inflammation, interstitial fibrosis, cardiomyocyte hypertrophy, myocyte apoptosis and necrosis, cardiac stiffness, arterial stiffness and increase of afterload (I stage)	Changed FFA metabolism, accumulation of AGEs (I stage)	LV mechanical impairment (II stage); LV diastolic dysfunction (III stage); HFpEF (V stage); HFrEF (VI stage)	LV hypertrophy (IV stage)	/	Conventional echocardiography and CMR (II and III stages)
Double clinical phenotypes		HFpEF phenotype	Hyperglycemia, lipotoxicity, hyperinsulinemia, and insulin resistance	Coronary microvascular endothelial inflammation, coronary microvascular stenosis, stiff and hypertrophied cardiomyocyte with a high resting tension	Accumulation of AGEs, impaired glucose metabolism, collagen deposition	LV diastolic dysfunction	Concentric LV remodeling, normal-sized, hypertrophied, and stiff LV	/	/
		HFrEF phenotype	Autoimmunity, lipotoxicity, reactive interstitial fibrosis, replacement fibrosis, loss of sarcomeres	Cardiomyocyte apoptosis, cardiomyocyte necrosis, coronary microvascular stenosis	Accumulation of AGEs and collagen deposition	Diastolic dysfunction	Eccentric LV remodeling, enlarged LV	/	/
A hypothesis		Subclinical hyperfunction	Impaired mitochondrial metabolism, activation of RAAS, impaired collagen crosslinking, loss of t-tubule structure, impaired Ca^2+^ sequestration of the sarcoplasmic reticulum, and oxidative stress	/	Formation of AGEs	Increased LV contractility and impaired diastolic function	Increased LV torsion and altered myocardial perfusion	/	Quantitative stress echocardiography

AT, angiotensin; AGEs, advanced glycation end products; CAN, cardiac autonomic neuropathy; CHD, coronary heart disease; CKD, chronic kidney disease; CTA, computed tomography angiography; CMR, cardiac magnetic resonance; EMB, endomyocardial biopsy; FFA, free fatty acid; GLUT, glucose transporter; HTA, arterial hypertension; HFpEF, heart failure with preserved ejection fraction; HFrEF, heart failure with reduced ejection fraction; IVUS, intravascular ultrasound; IGF, insulin-like growth factors; LGE, late gadolinium enhancement; MRI, magnetic resonance imaging; PCr/ATP, phosphocreatine/adenosine triphosphate; RAAS, renin-angiotensin-aldosterone system; ROS, reactive oxygen species; SNS, sympathetic nervous system; TGF-β, transforming growth factor beta.

Due to the origin of AHA and NYHA classification, the quartering model has strong practicability and popularity, which is definitely a good paradigm for examining DCM from a macro perspective and considering DCM complications. Six-division model is based on nuclear magnetic resonance, so it is more inclined to stage DCM from the perspective of imaging. If MRI is easy to obtain, this model is undoubtedly more refined, which is significant for clinical research. Unfortunately, although patients can be stratified, we lack the drugs to treat the lesions at each stage, particularly interstitial fibrosis, LV mechanical change, LVH, and HFpEF.

If DCM has two independent phenotypes, its value is enormous because it can save a lot of clinical resources to distinguish the stage and monitor the progression. The clinical test results of active cardiovascular drugs on HFpEF and HFrEF are different (64). However, the two-phenotype theory has a mixed reputation. LVSD is scattered in T2D. Most literature shows that resting LVEF in patients with T2D is normal. Therefore, whether T2D eventually develops into HFrEF is a controversial issue (9). Seferovic et al. proposed the hypothesis that T1D and T2D evolved respectively, which is convincing (64), but more research is required to confirm it. However, it was reported that LVEF in patients with relatively simple T2D at rest has a downward trend (194, 195). In fact, observational studies are mostly cross-sectional. Usually, only one of diastolic dysfunction and systolic dysfunction is included. Moreover, there is no prospective study to observe whether patients progress from diastolic to systolic dysfunction, so the evolution of DCM phenotype over time is still unknown. In addition, LVEF may improve or deteriorate during follow-up (196), and the measurement has a certain variability (197), which makes accurate classification difficult.

Notably, existing Spatio-temporal evolution models are mostly theoretical because the clinical diagnosis of DCM is challenging, so their data are mainly from experimental models (12). However, the proposed stages allow clinicians and researchers to see the whole picture. Moreover, the full spectrum of DCM clinical indicators will help optimize the evolution model and contribute to improvements in diagnosis since they all come from human studies.

### An overall picture of DCM

**Figure 2:**
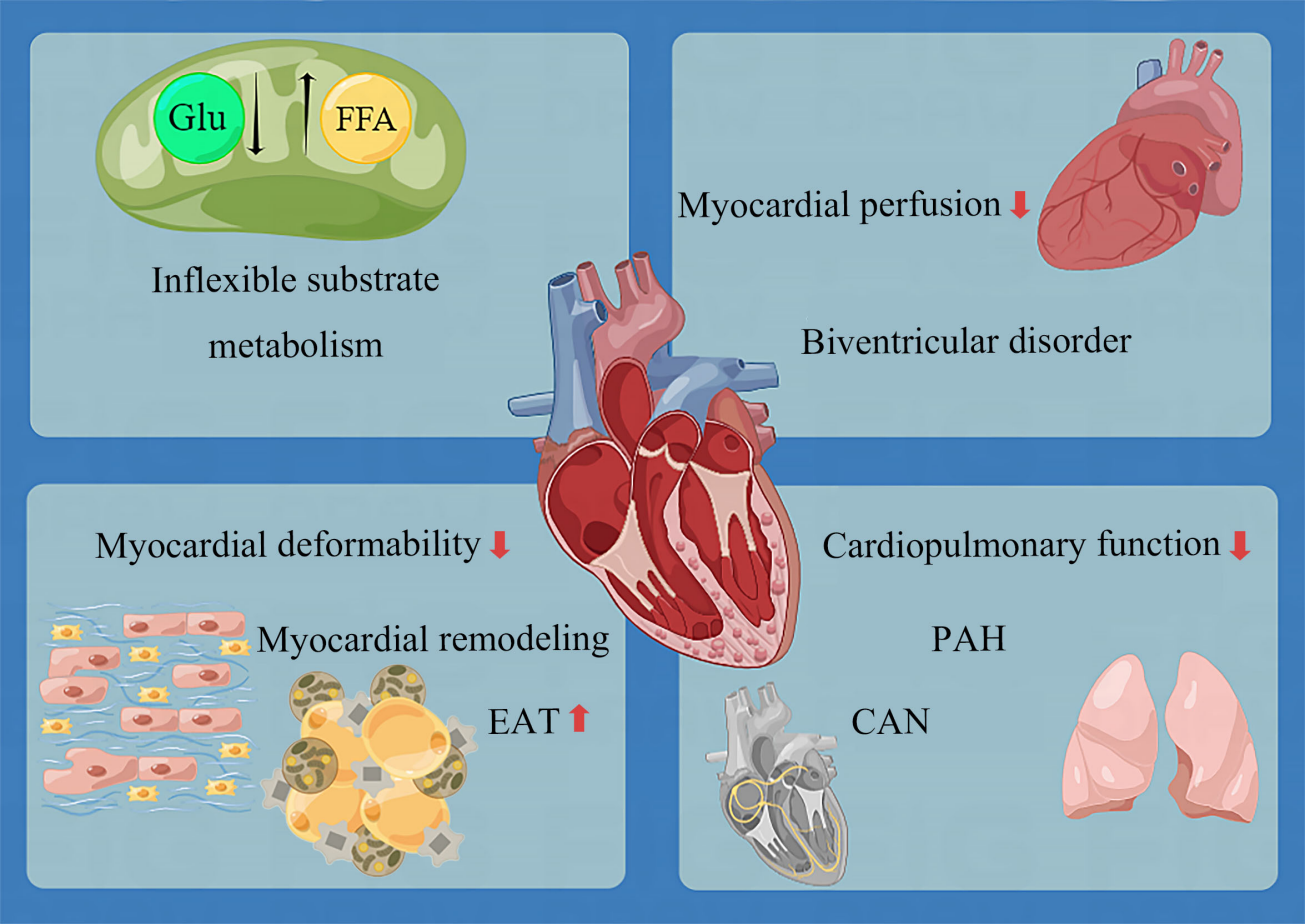
Overview of lesions in the development of DCM. CAN, cardiac autonomic neuropathy; EAT, epicardial adipose tissue; FFA, free fatty acid; Glu, glucose; PAH, pulmonary artery hypertension.

There is evidence that early in pre-diabetes, cardiometabolic recession appears alone (153, 158), followed by myocardial hypertrophy (148). Moreover, LVDD may appear. However, until the early stage of diabetes, heart damage remains tiny and discrete. As the disease progresses, the following phenomena occur (**Figure 2**):

- The myocardial energy metabolism substrates lose flexibility. Specifically, glucose uptake and utilization decrease, and fatty acid uptake and utilization increase, resulting in a large number of triglyceride deposition and a decrease in ATP production (144). Although the relationship between cardiac diastolic function and energy metabolism parameters is uncertain (30, 159), it has been confirmed that cardiac triglyceride deposition and myocardial energy damage are related to concentric LV remodeling and systolic dysfunction (138).
- Myocardial deformability decreases before diastolic dysfunction. Three layers of LV myocardium, including subendocardial (longitudinal fiber), medium (radial fiber), and subepicardial (circumferential fiber), are all damaged. Similar to the coronary ischemic myocardial injury (198), the damage to the subendocardial myocardium is the most serious, followed by the middle myocardium and the subepicardial myocardium (55). As for the left atrium, the function of all segments in the whole cardiac cycle is damaged (115). The unique feature of T1D is that the myocardial deformability of children is intact. The circumferential strain of the epicardium and middle layer and the longitudinal strain of the whole layer begins to decrease in late adolescence, followed by the decrease of conventional cardiac parameters years after (121).
- Remodeling involves in three chambers (left atrium, left and right ventricle), with systolic and diastolic function all impaired. LV remodeling is concentric hypertrophy characterized by an absolute and relative increase in LV wall thickness, increased LV mass (about 3.5 g) (136), and reduced LV cavity volume. RV remodeling is also restrictive, characterized by increased right ventricular stiffness and reduced right ventricular volume, similar to LV (143). In many diabetic patients, systolic and diastolic dysfunction often go hand in hand (103). In severe cases, LVEF and RVEF decreases.
- The epicardial adipose tissue becomes thickened.
- Myocardial perfusion decreases in both coronary artery and microvessels. This change is sufficient to be detected at rest and more obvious at stress (dobutamine and exercise).
- Cardiopulmonary function decreases, manifested as a decrease in myocardial oxygen uptake, and increase in oxygen consumption.

Except these alterations, the pulmonary artery adjacent to the RV is not spared. As early as 2005, the link between T2D and PAH (Pulmonary Artery Hypertension) had been demonstrated by an epidemiological study, showing an increased risk of PAH in diabetic patients independent of smoking, coronary heart disease, hypertension, or congestive heart failure (199). PAH is a disease characterized by pulmonary vascular remodeling, which accelerates the pace of right heart failure by increasing the right ventricular afterload. Insulin resistance, frequently found in diabetic patients with increased pulmonary artery pressure, increases pulmonary stiffness and decreases pulmonary elasticity (48). The prognosis of PAH is poor, and the 10-year survival rate of PAH patients with diabetes is worse (200). DCM may be a cardiopulmonary disease to some extent. Unfortunately, interventions to improve RV function and prognosis in patients with DCM are yet to be studied.

DCM eventually develops into biventricular disorder (48, 201). Although the original research data on T1D are not as detailed as those of T2D, we can find that the cardiac phenotype changes of T1D and T2D are approximately the same. The only noteworthy finding was evidence of cardiac hyperdynamic contraction in T1D but not T2D; this may be attributed to the fact that the course of disease of patients included in T2D research is generally too long, and the opportunity to find this phenomenon is missed. In addition, only sporadic studies have focused on the exact changes in the heart of patients with pre-diabetes. More data accumulation is still needed to understand the impact of pre-diabetes on the heart.

Some scholars believe that DCM affects the whole heart (46). However, we are temporarily unable to decide the situation of right atrium (RA). Although some studies (202–204) found that DM causes RA volume expansion and systolic and diastolic function decline, the subjects were mixed with CAD or hypertension patients, making accurate judgments difficult. However, we confirm that the impact of diabetes on the heart will damage the heart metabolism, structure, function, perfusion, epicardium, and pulmonary vascular, which is comprehensive. In addition to the changes in the heart itself, DM will also cause a series of hematological changes, mainly on the entry of information substances secreted by the heart and other tissues into the plasma.

Another fact is that the pathogenesis of DCM includes CAN (14), making it difficult to separate these two diseases. There is evidence that in T2D patients with normal blood pressure, CAN precedes LVH and diastolic dysfunction, which is a very early change in the heart of DM (205). Furthermore, CAN is gradually aggravated parallel to the progression of cardiomyopathy. Because of the close relationship between nerve and muscle in the heart, the extent of CAN participation in DCM is worth exploring because it is related to the essence of DCM, either nerve-muscle or simple muscle disease.

### Diagnostic marker

#### ECG

Changes in myocardial electrophysiology can be found in DCM patients, such as the prolongation of QT interval, the increase of QT dispersion, and T peak-Tend dispersion. These repolarization abnormalities represent the asynchronous myocardial movement and have been reported to reflect LVDD (130). However, ECG is not specific, making it difficult to diagnose DCM simply by ECG.

#### Serum marker

Some new markers appear to be promising for use in clinical diagnosis and treatment. Fibrotic markers play a major role. The increase of active MMP-9 and MMP-7 and the decrease of TIMP-1/active MMP-9 ratio have been detected in DM-DD (diastolic dysfunction) (82). It was also found that serum PIP level was negatively correlated with A-Ar (estimated passive diastolic function) in early T2D, suggesting that fibrosis might indeed be the cause of diastolic dysfunction (83). Some other markers have also been studied. GDF-15 is a stress response cytokine that is elevated in asymptomatic DCM and significantly associated with E/e’ (diastolic function index) (80). IGFBP-7 is expected to be a marker for DCM as it increased progressively in patients with DM, DM-DD, and DM-SD (systolic dysfunction) and did not increase in DD patients without diabetes (79). Another study confirmed that NLR was positively correlated with impaired LVDD and was an independent risk factor for subclinical DCM (77), but NLR is also well correlated with other diabetic complications (206). In addition, some crucial biomarkers deserve attention: FABP3, activin A, CT-1, YKL40, galectin-3, and FGF21. They are involved in various pathological mechanisms and have good early diagnostic potential (207).

In recent years, non-coding RNA has been developing rapidly in the field of diagnosis, but many original studies have focused on DM, and there are few studies on DCM markers (208). miRNAs are small non-coding RNAs responsible for post-transcriptional regulation of gene expression. miRNAs can be isolated from tissues and body fluids, and its level can be detected by qPCR, *in situ* hybridization array, and RNA sequencing (209). Two DCM-related miRNAs (**Table 1**) have been found. It is striking that one study seems to have found a specific lncRNA marker for DCM. Researchers reported that lncRNA NKILA was up-regulated in the plasma of DCM patients but not in patients with other complications (87). Plasma lncRNA NKILA mRNA levels six months before diagnosis were sufficient to screen DCM in DM patients. Eight years of follow-up research revealed that the expression of the lncRNA NKILA was specifically up-regulated when DCM was present. LncRNA NKILA is a molecule that promotes cardiomyocyte apoptosis, which may be involved in the occurrence and development of DCM and has a bright future in diagnosis or treatment (87).

Some argue that DCM cannot be diagnosed by a single serological marker, so a combination may be needed to solve this problem. Some researchers had begun to endeavor. A study showed that the AUCs of IL-6, TNF-α, and AGEs were 0.905, 0.845, and 0.807, respectively, which could not diagnose DCM. However, the combination of these biomarkers significantly increased AUC to 0.924, with a sensitivity of 84.8% and a specificity of 88.2% (79). The union of TNF-α, AGEs, creatinine, and insulin helped diagnose DM-DD (AUC 0.913, specificity 100%) (79). Nevertheless, the combination of IL-6 and AGEs was found helpful for further differential diagnosis of DM-SD and DM-DD, with an AUC of 0.795 and sensitivity of 90.6%, which was significantly better than that of a separate diagnosis (79).

#### Echocardiogram

In recent years, LV GLS has become the most commonly used strain value (117) for evaluating LV function in diabetic patients. GLS damage is likely the first ultrasonic sign of preclinical diabetic cardiomyopathy, confirmed in both T1D and T2D (98, 103). In theory, longitudinal myocardial fibers are more prone to ischemia and fibrosis because compensatory ventricular remodeling may increase short-axis function (210). A meta-analysis showed that (126) three-dimensional GLS was 2.4% lower in diabetic patients than in healthy controls, and it is the most obvious indicator of three-dimensional LV systolic strain in all directions. GLS can also be used to exclude DCM. The detection of NPV is up to 0.94 when joined with Gal-3 (81). It should not be ignored that LA strain has changed before LV strain changes, so as an early parameter, it may be more sensitive than LV strain for detecting early DCM (133); this is because LA is a fragile monolayer wall and is sensitive to subtle stimuli (211).

#### Cardiac magnetic resonance

CMR is accurate for myocardial imaging. Furthermore, one-stop detection of DM heart may be realized in the future due to its excellent differential diagnosis ability of coronary atherosclerotic heart disease and lack of need for a contrast agent (212).

The participation of myocardial microvascular dysfunction in DCM remains uncertain because even ischemia has been excluded, obese patients are inevitably mixed in T2D, and it can also aggravate the adverse effect of diabetes on microvascular function. However, a new study has confirmed that, rather than BMI, HbA1c is the only independent risk factor for myocardial microvascular function in T2D patients (145). Myocardial microvascular dysfunction begins in the early stage of T2D and accumulates with the course extension. A CMR study has reported upslope, Max SI (max signal intensity), and TTM (time to maximum signal intensity) changes, which are indicators of coronary microcirculation impairment in T2D. Multivariate regression analysis shows that TTM and upslope are independently associated with longitudinal PSSR (peak systolic strain rate), suggesting that there might be a mechanical linkage between myocardial perfusion impairment and subclinical myocardial dysfunction in T2D patients (57).

As a result of the application of T1 mapping technology, interstitial fibrosis becomes easy to measure. The more severe interstitial fibrosis is, the worse LV diastolic function becomes (152). ECV can measure dilated ECM (extracellular matrix), so ECV can reflect myocardial fibrosis when there is no myocardial edema or protein deposition (150). The ECV value of diabetic patients has been proven significantly increased, especially those with poor blood glucose control (150). So it may be a sensitive parameter to detect early remodeling before dysfunction. A study between obese adolescents and healthy volunteers found that increased ECV occurred parallel with changes in LV mass and volume, but LV function remained unchanged (151).

As for T1D, researchers introduced a new CMR marker, DRF indice, which can detect ventricular diastolic efficiency and dynamic changes in the diabetic heart between 16-21 years old so that it may be a sensitive marker of cardiac dysfunction in adolescents (141). However, the study of CMR in T1D patients is still lacking.

The diagnostic marker of DCM should be a humoral or structural/functional indicator directly related to its pathogenesis and has good exclusiveness. Unfortunately, no mature auxiliary examination marker has been reliably tested in large clinical trials to diagnose DCM up to this point.

### Clinical treatment

#### hypoglycemic drugs

For DCM treatment, people’s first consideration is whether the hypoglycemic drugs can inhibit the process of impaired cardiac function in DM patients. However, the results are still a little far from satisfaction. Among conventional oral antidiabetic drugs, only gliclazide significantly reduced LVM (213), and their cardiac benefits are tiny (14).

Some scientists focused on DPP4i (sitagliptin, alogliptin, and saxagliptin). All three agents have been evaluated in large clinical trials, but they did not affect the composite primary outcome, which included cardiovascular (CV) mortality, nonfatal MI, and nonfatal stroke (214). As for the influence on DCM, Sitagliptin had a better hypoglycemic effect than voglibose, but it did not improve LV function in 24 weeks of treatment (178). While years later, another study showed that the addition of sitagliptin to the conventional hypoglycemic regimen for 24 months alleviated LVDD (179).

GLP-1RA (liraglutide, exenatide, semaglutide, lixisenatide, and dulaglutide) has attracted much attention as a new star, because it has been shown to have CV protective effects as well as weight loss (215), but its performance is mediocre in DCM. Liraglutide is an injection drug given once a day, whose 3-point MACE (major adverse CV events) benefits has been proved by LEADER trial (216). Two studies have shown that early application of liraglutide (for about six months) improved cardiac function (187, 188), and Liraglutide is more effective than sitagliptin and linagliptin, both in terms of improving diastolic function and proteinuria (217). While another study has shown that the exact duration of liraglutide treatment did not improve cardiac function in patients (189). As for once-weekly Exenatide, who showed no influence on 3-point MACE (218), a study showed that it can improve cardiac function and reduce arterial stiffness, but it is not accompanied by an improvement in exercise capacity (185). However, there is also evidence that exenatide does not affect cardiac function, perfusion, or oxidative metabolism in T2D patients with LVSD (186). Despite Liraglutide seems a little better than Exenatide in CV effect, its frequent injections and increase of heart beats should not be ignored (219). But in any case, these drugs have a positive effect on coronary atherosclerotic events (215). Although not all five GLP-1RA have CV benefits, their CV safety is indisputable, so they are still worthy of consideration in obese DCM patients with mixed atherosclerotic disease.

Of note, SGLT-2i, which includes Dapagliflozin, Empagliflozin and Canagliflozin, have all been proved to help decrease the rate of CV events or heart failure and even death (220–222). When it comes to DCM, Dapagliflozin can prevent and reverse the development of ventricular remodeling (180, 181). The efficacy of Empagliflozin is diverse. 12 weeks’ empagliflozin treatment not only enhances cardiac function, attenuates adverse remodeling, but also improves myocardial energy metabolism, which is not found in the other two (184). And another study showed that the earlier the intervention implements, the better the improvement in left ventricular diastolic and systolic function will be (183). There is a study which has shown that 3 months of canagliflozin treatment can improve left ventricular diastolic function, but it should be noted that the study population was mixed with ischemic heart disease patients, limiting the reference value (182).

Besides, both Dapagliflozin and Canagliflozin showed significant renal benefits (220, 221). It is highly likely that DCM is associated with renal injury because microalbuminuria is associated with early DCM as mentioned above, which makes these two drugs worthy of further investigation. By the way, a meta-analysis showed that the efficacy of SGLT-2i was sex specific, which reduces MACE better in men than in women. However, GLP-1RA did not show this difference (223). In general, SGLT-2i is more promising than GLP-1RA in DCM. Because the former has better performance in preventing heart failure and improving renal outcomes while similar to the latter in atherosclerotic benefits, and is injection-independent (224). In addition to this, GLP-1RA on top of SGLT-2i is a hopeful direction. Preliminary studies have shown that the combination of liraglutide and empagliflozin for 12 months significantly improves effective myocardial work and cardiac function in patients with type 2 diabetes mellitus in high CV risk. However, similar work needs to be done in patients excluding coronary heart disease and hypertension to determine whether the effect persists.

#### Cardiovascular drugs

Although conventional cardiovascular drugs (ACEI, ARB, and β blocker) are widely used in the diabetic population, there is a lack of evidence regarding the clinical efficacy of DCM. But new heart failure drugs are likely to play an important role in DCM induced HFrEF in the near future. Large studies have shown that patients with combined use of new drugs (ARNi, β-blocker, MRA and SGLT2i) can delay 2.7 years (80 years) to 8.3 years (55 years) without death from cardiovascular disease or first hospitalization for heart failure, and increase life expectancy by 1.4 years (80 years) to 6.3 years (55 years), compared with conventional therapies (225). Notably, three large cohorts each had more than 30% of patients with DM, heralding a promising future for these agents in DM.

Sacubitril/valsartan inhibits neprilysin through its active metabolite of sacubitril, and at the same time blocks angiotensin II type 1 receptor through valsartan, which plays an anti-heart failure role. Sacubitril/valsartan reduced NT-proBNP to a greater extent than valsartan at 12 weeks and was well tolerated in patients with HFpEF (226). And the benefit of Sacubitril/valsartan is across all age groups from 18 to 96 years old (227). However, in patients with HFpEF (EF ≥45%), sacubitril/valsartan did not significantly reduce heart failure hospitalizations or cardiovascular mortality (228). Unexpectedly, the investigators also found a beneficial effect of sacubitril/valsartan on glycemic control. In patients with diabetes and HFrEF enrolled in the PARADIGM-HF study, long-term reductions in HBA1C and delayed time to initiation of oral glucose-lowering therapy were observed (229).

Vericiguat, approved by the U.S. FDA in 2021, is the first soluble guanylate cyclase (sGC) agonist for the treatment of patients with symptomatic HFrEF. Patients with chronic HFrEF who received vericiguat had a lower incidence of cardiovascular death or hospitalization for heart failure than those who received placebo (230). However, in patients with decompensated HFpEF, vericiguat treatment for 24 weeks did not improve activity tolerance and quality of life which were measured by KCCQ (231). The safety and tolerability profile of vericiguat were good when sacubitril/valsartan was used together for at least 3 months, suggesting that the combination may provide additional benefit (232).

Elevated resting heart rate is a risk factor for poor outcomes in heart failure (233). when heart rate is ≥70 beats/min, the risk of cardiovascular outcomes increases in patients with CAD and LVSD (234). This sparked scientists’ interest in Ivabradine, a drug that lowers the heart rate. Ivabradine was shown to be effective and safe in patients with HFrEF regardless of diabetes status, and significantly reduced the primary composite endpoint (PCE) in both diabetic and non-diabetic patients (235). However, a randomized, double-blind, placebo-controlled trial found that heart rate reduction with ivabradine did not improve outcomes in HFpEF patients (236).

Omecamtiv mecarbil is the first potent cardiac myosin activator that can specifically activate the cardiac myosin S1 structure. A preclinical report has shown that it increases myocardial contractility without increasing myocardial oxygen consumption (237), and in clinical trials, it improved the composite rate of heart failure events or death from cardiovascular causes (238). The improvement of cardiac function by Omecamtiv was also very significant. Omecamtiv treatment for 20 weeks directly improved LV myocardial deformation in patients with HFrEF, whch means improved GLS by about 0.8%, and also improved GCS, while reducing heart rate and NT-proBNP concentrations, significantly increasing systolic ejection time, stroke volume, and reversing LV remodeling have been reported (239). Each 1% improvement in GLS is statistically associated with a 24% reduction in the risk of HF hospitalization and death (240). What’s more, omecamtiv also results in a small reduction in heart rate (241). Unfortunately, omecamtiv did not improve exercise capacity compared with placebo over a period of 20 weeks in patients with chronic HFrEF (242).

Other than those, some non-classical cardiovascular drugs appear to show some different potential. The critical role of inflammatory cytokines in developing cardiovascular disease in individuals with and without diabetes is well established. A study has shown that trimetazidine treatment could significantly improve cardiac function and physical tolerance, accompanied by a reduction in inflammation (193), but it could not explain if there is a link between inflammation and the outcome. Another study found that sildenafil could control DCM progression by targeting IL-8 at the systemic and cellular levels (192). A previous study had also shown that sildenafil could achieve its anti-remodeling effect by reducing LV concentric hypertrophy in patients with early DCM (191). In addition, an aldosterone receptor antagonist, Spironolactone, has been proven to be failed to improve DCM’s changes in myocardial structure and diastolic function (243).

**Figure 3:**
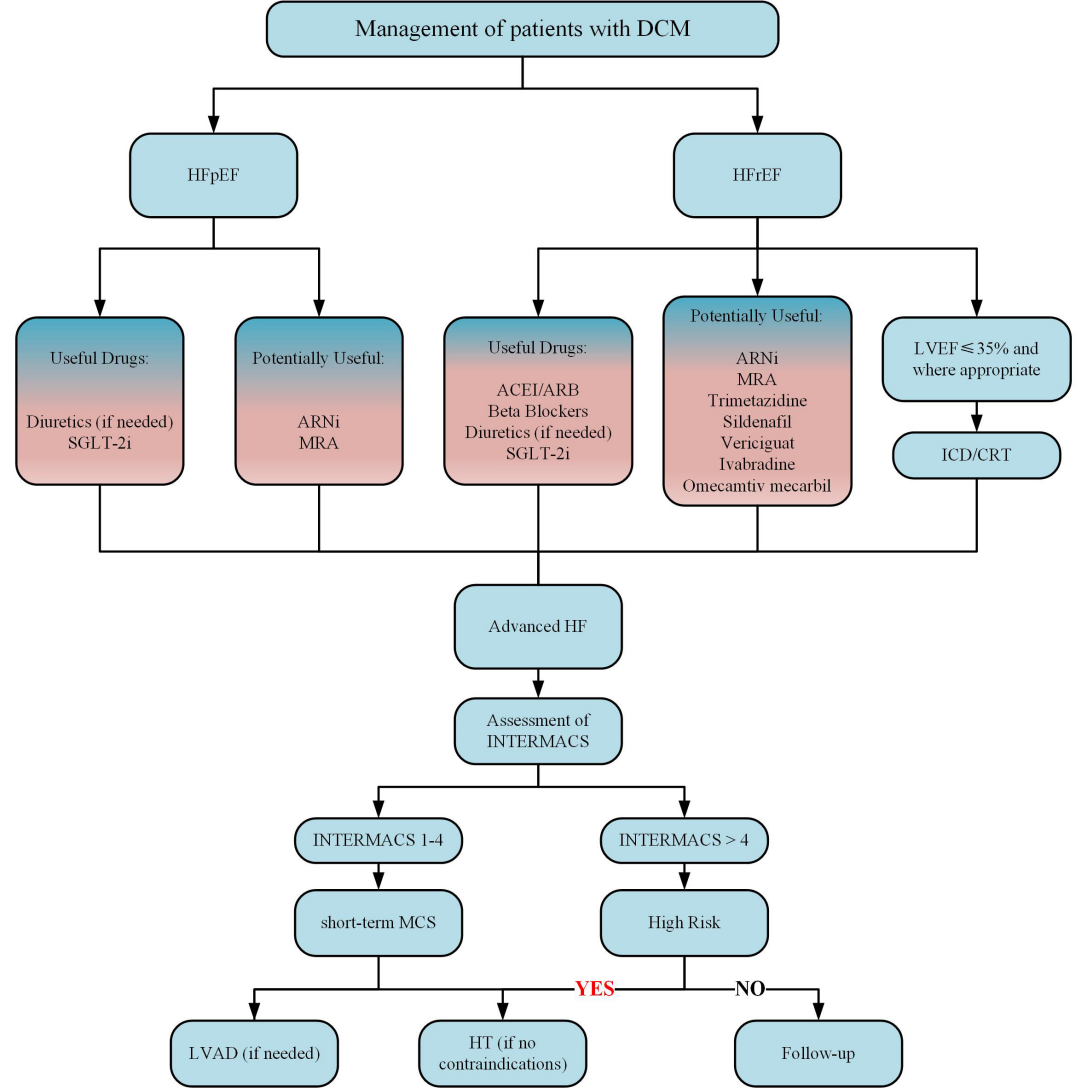
Treatment algorithm of DCM. ACEI, indicates angiotensin-converting enzyme inhibitor; ARB, angiotensin receptor blocker; ARNi, angiotensin receptor-neprilysin inhibitor; CRT, cardiac resynchronization therapy; HF, heart failure; HFrEF, heart failure with reduced ejection fraction; HFpEF, heart failure with preserved ejection fraction; HT, heart transplant; ICD, implantable cardioverter-defibrillator; INTERMACS, Interagency Registry for Mechanically Assisted Circulatory Support; LVAD, left ventricular assist device; MCS, mechanical circulatory support; MRA, mineralocorticoid receptor antagonist; SGLT2i, sodium-glucose cotransporter 2 inhibitor.

According to the existing drug research results and HF guidelines (244, 245), we summarized a therapeutic algorithm (**Figure 3**). To date, neither the long-term survival problem caused by HFpEF nor the limitation of exercise in HF patients can be improved by any drug. To make matters worse, no drug independently treats DCM as a primary role.

## Conclusion

As a complication of DM, DCM has been studied for 50 years. The vast accumulation of knowledge has painted the outline of DCM so humans can overlook the panorama of clinical phenotypes. More observational clinical studies are needed to continuously improve the undiscovered corners of DCM, correct previous studies’ results, and explore indicators that can be used for accurate diagnosis. Screening criteria still need to be further defined, which is vital for finding high-risk populations of DCM. Nevertheless, it is gratifying that the diagnostic criteria have been established, which allows the real focus on implementing clinical intervention studies of DCM, even if the threshold is high. The accumulation of DCM research is significant. When achieving development of safe and effective diabetic cardiomyopathy drugs, whether combined with diabetes will affect the classification, diagnosis, and treatment of HF and change the dilemma that the prognosis of HF patients with diabetes is worse than that of HF patients alone. And the dawn will eventually come.

## Author contributions

XZ: Writing- Original draft preparation. SL: Writing- Reviewing and Editing. GF: Conceptualization, Methodology. BW: Supervision. XW: Software, Visualization. SW: Funding acquisition. PP, QY, SD and JL: Data curation. YC: Methodology. All authors contributed to the article and approved the submitted version.
